# *Eleutherococcus sessiliflorus* Inhibits Receptor Activator of Nuclear Factor Kappa-B Ligand (RANKL)-Induced Osteoclast Differentiation and Prevents Ovariectomy (OVX)-Induced Bone Loss

**DOI:** 10.3390/molecules26071886

**Published:** 2021-03-26

**Authors:** Sang-Yong Han, June-Hyun Kim, Eun-Heui Jo, Yun-Kyung Kim

**Affiliations:** 1 Department of Herbal Medicine, College of Pharmacy, Wonkwang University, 460 Iksandae-ro, Iksan, Jeonbuk 54538, Korea; 030745@daum.net; 2 Wonkwang Oriental Medicines Research Institute, Wonkwang University, 460 Iksandae-ro, Iksan, Jeonbuk 54538, Korea; 3 Department of Acupuncture & Moxibustion Medicine, College of Korean Medicine, Wonkwang University, 460 Iksandae-ro, Iksan, Jeonbuk 54538, Korea; wnsgus05@gmail.com; 4 Research Center of Traditional Korean Medicine, Wonkwang University, 460 Iksandae-ro, Iksan, Jeonbuk 54538, Korea

**Keywords:** *Eleutherococcus sessiliflorus*, Osteoporosis, RANKL, Osteoclast differentiation, NFATc1, c-Fos, Bone loss

## Abstract

The aim of this study was to evaluate the effects of root bark of *Eleutherococcus sessiliflorus* (ES) on osteoclast differentiation and function in vitro and in vivo. In vitro, we found that ES significantly inhibited the RANKL-induced formation of TRAP-positive multinucleated osteoclasts and osteoclastic bone resorption without cytotoxic effects. ES markedly downregulated the expression of nuclear factor of activated T cells cytoplasmic 1 (NFATc1); c-Fos; and osteoclast-related marker genes, such as TRAP, osteoclast-associated receptor (OSCAR), matrix metalloproteinase-9 (MMP-9), calcitonin receptor, cathepsin K, the 38 kDa d2 subunit of the vacuolar H^+^-transporting lysosomal ATPase (Atp6v0d2), dendritic cell-specific transmembrane protein (DC-STAMP), and osteoclast-stimulatory transmembrane protein (OC-STAMP). These effects were achieved by inhibiting the RANKL-mediated activation of MAPK signaling pathway proteins, including p38, ERK, and JNK. In vivo, ES attenuated OVX-induced decrease in bone volume to tissue volume ratio (BV/TV), trabecular thickness (Tb.Th), trabecular number (Tb.N), and bone mineral density, but increased trabecular separation (Tb.Sp) in the femur. Collectively, our findings showed that ES inhibited RANKL-activated osteoclast differentiation in bone marrow macrophages and prevented OVX-mediated bone loss in rats. These findings suggest that ES has the potential to be used as a therapeutic agent for bone-related diseases, such as osteoporosis.

## 1. Introduction

Osteoporosis is a systemic skeletal disease characterized by a decrease in bone mass and abnormalities in the bone microstructure, resulting in an increased risk of fractures. It mainly occurs in postmenopausal women and elderly men. Due to the aging of the population, the incidence of osteoporosis is increasing every year [1,2]. The treatment for osteoporosis includes two types of therapies: antiresorptive and anabolic. Antiresorptive drugs primarily reduce bone resorption, while anabolic drugs increase new bone formation. Currently, the known antiresorptive agents include bisphosphonates, selective estrogen receptor modulators, estrogens, calcitonin, and monoclonal antibodies, while anabolic drugs while anabolic drugs include the teriparatide (parathyroid hormone (PTH) 1-34), abaloparatide (PTH-related protein (PTHrP) analog), and romosozumab [3,4]. Several therapeutic agents for osteoporosis increase bone mineral density (BMD) and reduce the risk of skeletal fractures; however, these treatments were reported to have various adverse effects, such as osteonecrosis of the jaw, thrombosis, atypical femur fractures, hypocalcemia, and stroke [3,4,5].

Medicinal plants and foods provide an opportunity to discover effective and safe treatments for bone-related diseases. Many studies have reported that various crude plant extracts prepared from medicinal plants and foods have anti-osteoporosis effects by inhibiting osteoclast differentiation [6,7,8] and enhancing osteoblast formation [9,10].

*Eleutherococcus* spp. is also called *Acantopanax* spp. In *Eleutherococcus* spp., there are several species used as herbs. Five local variants—*Eleutherococcus sessiliflorus* (Rupr. & Maxim.) S.Y.Hu., *Eleutherococcus nodiflorus* (Dunn) S.Y.Hu., *Eleutherococcus giraldii* (Harms) Nakai, *Eleutherococcus henryi* Oliv., and *Eleutherococcus verticillatus* (G.Hoo) H.Ohashi—are used as ogapi (五加皮) [11]. On the other hand, *Eleutherococcus senticosus* (Rupr. & Maxim.) Maxim. is used as jaoga (刺五加), which is well known and used frequently in Russia [12]. These two herbs are separated in Korean and Chinese pharmacopoeia [13,14], and both are listed in the European Pharmacopoeia [15]. However, USA dietary supplements compendium recorded Eleuthero (*Eleutherococcus senticosus* (Rupr. & Maxim.)) only [16].

*Eleutherococcus sessiliflorus* (Rupr. & Maxim.) S.Y.Hu. belongs to the family Araliaceae. Several *Eleutherococcus* species have been shown to have various biological effects, including anti-inflammation, anticancer, antioxidant, and anti-obesity [17]. Many studies have identified the chemical constituents of *Eleutherococcus* species, which include phenylpropanoids, lignins, sterols, coumarins, vitamins, minerals, and mono- and polysaccharides [17]. The dried root bark of *Eleutherococcus sessiliflorus* (Rupr. & Maxim.) S.Y.Hu. (ES) is known as ogapi in the Korean Pharmacopoeia [18]. It is traditionally used in Korean medicine as an important medication and is described to “expel wind and damp, nourish the liver and kidney, strengthen bones and tendons” [19]. ES has been reported to exhibit various pharmacological activities, including anti-inflammatory [20], anti-osteoporotic [21], antitumor [22,23] anti-oxidative, and antiaging [24] effects. In the present study, we investigated the anti-osteoporotic effects of ES on RANKL-induced osteoclast differentiation in bone marrow macrophages (BMMs) and on bone destruction in OVX-induced bone loss animal models.

## 2. Results

### 2.1. High-Performance Liquid Chromatography (HPLC) Analysis of ES Water Extracts

**Figure 1:**
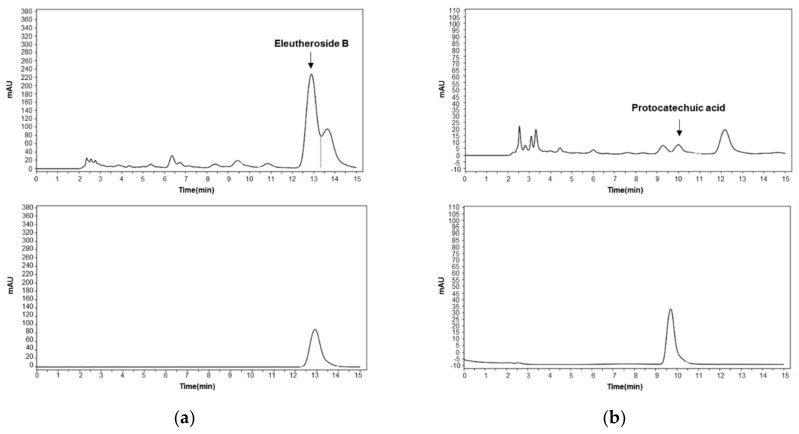
HPLC for the qualitative analysis of eleutheroside B and protocatechuic acid in the *Eleutherococcus sessiliflorus* (ES) extracts. (**a**) The chromatograms for ES (upper) and standard substance eleutheroside B (lower). (**b**) The chromatograms for ES (upper) and standard substance protocatechuic acid (lower).

Two compounds of ES water extracts were identified by HPLC analysis: eleutheroside B and protocatechuic acid (Figure 1). Eleutheroside B and protocatechuic acid were detected at a retention time of a 12.889 min and 9.986 min, and concentration of 0.0542 mg/mg and 0.0013 mg/mg, respectively.

### 2.2. ES Inhibits RANKL-Induced Osteoclastogenesis

**Figure 2:**
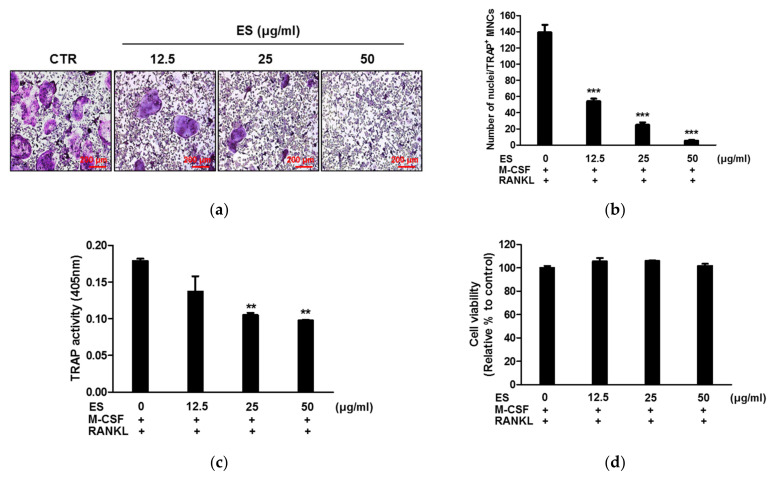
*Eleutherococcus sessiliflorus* (ES) suppressed receptor activator of nuclear factor kappa-B ligand (RANKL)-mediated osteoclast differentiation. Bone marrow macrophages (BMMs) were treated with ES (12.5, 25, and 50 μg/mL) in the presence of M-CSF (30 ng/mL) and RANKL (100 ng/mL) for 4 days. (**a**) BMMs were stained for the TRAP solution. Images were photographed under a light microscope (100× magnification). (**b**) Number of nuclei per TRAP-positive multinucleated cells were counted as osteoclasts (*n* = 5). (**c**) TRAP activity was determined by measuring OD at 450 nm. (**d**) Cell viability was assessed by the XTT assay. All data were confirmed by technical replicated (*n* = 3). The data presented are the mean ± S.D. of three independent experiments. ** *p* < 0.01, *** *p* < 0.001 vs. the control.

To examine the effects of ES on RANKL-induced osteoclastogenesis, BMMs treated with M-CSF and RANKL were cultured in the presence or absence of ES for 4 days. Figure 2a shows that RANKL treatment increased, and ES treatment decreased, the formation of TRAP-positive multinucleated osteoclasts. Consistent with these morphological changes, ES significantly decreased the number of TRAP-positive multinucleated osteoclasts and the activity of TRAP (Figure 2b,c). To determine the cytotoxicity of ES, BMMs were treated with various concentrations of ES (12.5, 25, and 50 μg/mL) in the presence of M-CSF, and cell viability was measured by the XTT assay. ES did not cause any cytotoxic effects at any tested concentration (Figure 2d).

### 2.3. ES Inhibits Actin Belts Formation and Bone Resorption in Osteoclasts

**Figure 3:**
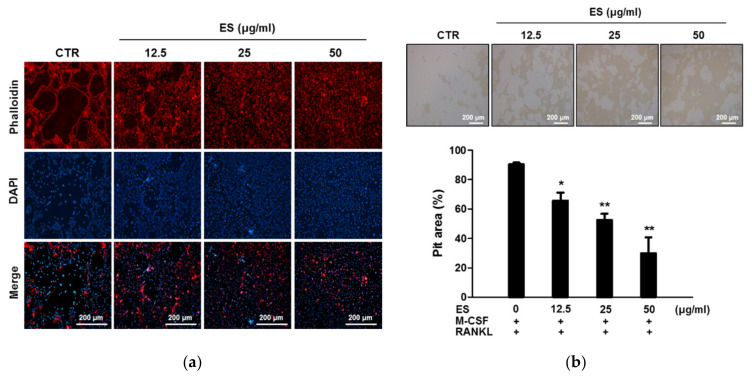
ES suppressed F-actin belts formation and bone resorption. (**a**) Osteoclasts were treated with or without ES for 4 days and then stained with Texas Red phalloidin. (**b**) Mature osteoclasts were seeded on mineralized matrix Osteo Assay Surface 96-well plates and treated with ES (12.5, 25, and 50 μg/mL) for 24 h. The cells were removed and photographed under a light microscope. The percentages of resorption pit areas were quantified using ImageJ 1.50i (right). * *p* < 0.05, ** *p* < 0.01 vs. the control.

The formation of F-actin belts in mature osteoclasts is one of the targets for the control of bone resorption. Even though ES could inhibit osteoclastogenesis, it was unclear whether ES had a direct effect on F-actin belts formation and bone resorption. Therefore, first, we performed an F-actin belts assay using phalloidin staining. As shown in Figure 3a, formation of F-actin belts was evidently increased in the control group. However, ES completely disrupted the number and size of F-actin belts. Next, we examined whether ES inhibited RANKL-activated bone resorption by measuring pit formation assay. As a result, bone resorption was activated by RANKL stimulation. The number and area of bone resorption were substantially decreased by ES treatment in a concentration-dependent manner (Figure 3b). These results suggested that ES inhibited bone resorption pits and F-actin belts formation.

### 2.4. ES inhibits RANKL-Induced c-Fos and NFATc1 and Osteoclast-Specific Gene Expression

**Figure 4:**
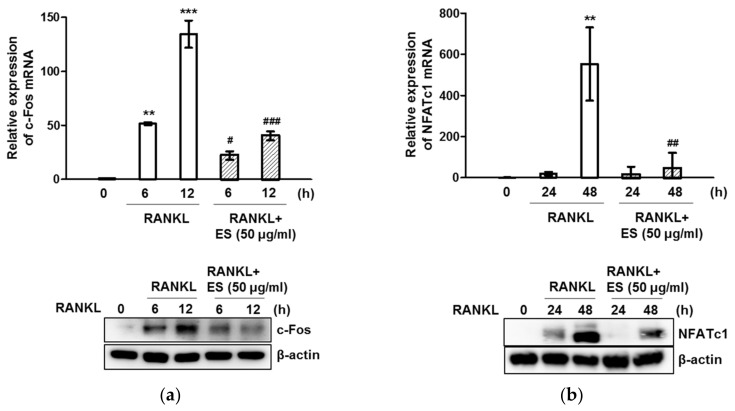
ES suppressed RANKL-mediated expression of c-Fos and nuclear factor of activated T cells cytoplasmic 1 (NFATc1). BMMs were stimulated with or without 50 μg/mL ES in the medium containing M-CSF (30 ng/mL) and RANKL (100 ng/mL) for the indicated periods. (**a**,**b**, top) The mRNA expression of c-Fos and NFATc1 were analyzed by real-time PCR. GAPDH was used as the internal control. (**a**,**b**, bottom) The protein expression was evaluated using western blot analysis. β-Actin was used as the internal control. The results are presented as mean ± S.D. of three independent experiments. ** *p* < 0.01, *** *p* < 0.001 vs. the control; ^#^ *p* < 0.05, ^##^ *p* < 0.01, ^###^ *p* < 0.001 vs. the control at each corresponding time point.

**Figure 5:**
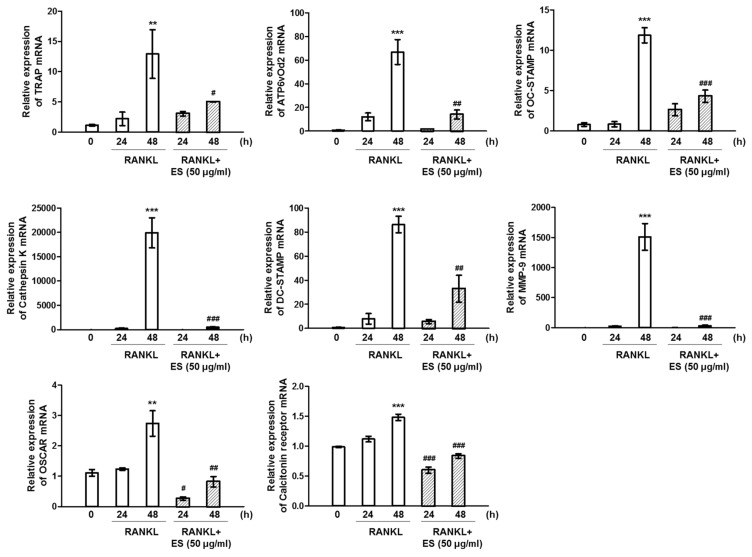
ES suppressed the expression of osteoclast-specific genes. BMMs were treated with or without ES (50 μg/mL) in medium containing M-CSF (30 ng/mL) and RANKL (100 ng/mL) for the indicated time points. The mRNA levels of TRAP, osteoclast-associated receptor (OSCAR) 38 kDa d2 subunit of the vacuolar H+-transporting lysosomal ATPase (Atp6v0d2), osteoclast-stimulatory transmembrane protein (OC-STAMP), dendritic cell-specific transmembrane protein (DC-STAMP), calcitonin receptor, matrix metalloproteinase-9 (MMP-9), and cathepsin K were determined by real-time PCR. The results are the expressed as the mean ± S.D. of three independent experiments. ** *p* < 0.01, *** *p* < 0.001 vs. the control at 0 h; ^#^ *p* < 0.05, ^##^ *p* < 0.01, ^###^ *p* < 0.001 vs. control at each corresponding time point.

To elucidate the effect of ES on RANKL-induced osteoclast formation and function, we examined the expression of osteoclast-specific genes. Quantitative RT-PCR analysis indicated that RANKL stimulation upregulated c-Fos and NFATc1 mRNA levels, whereas ES considerably diminished the RANKL-activated induction of both transcription factors (Figure 4a,b, top). Western blot data showed that ES reduced protein levels of c-Fos and NFATc1 in a time-dependent manner (Figure 4a,b, bottom). The presence of RANKL also increased TRAP, Atp6v0d2, OSCAR, calcitonin receptor, OC-STAMP, DC-STAMP, MMP-9, and cathepsin K mRNA levels, whereas the ES treatment markedly suppressed the expression of these genes (Figure 5). These results suggest that ES may inhibit osteoclast formation and function by suppressing RANKL-activated osteoclast-specific genes.

### 2.5. ES Inhibits Osteoclastogenesis via MAPK and NF-κB Signaling

**Figure 6:**
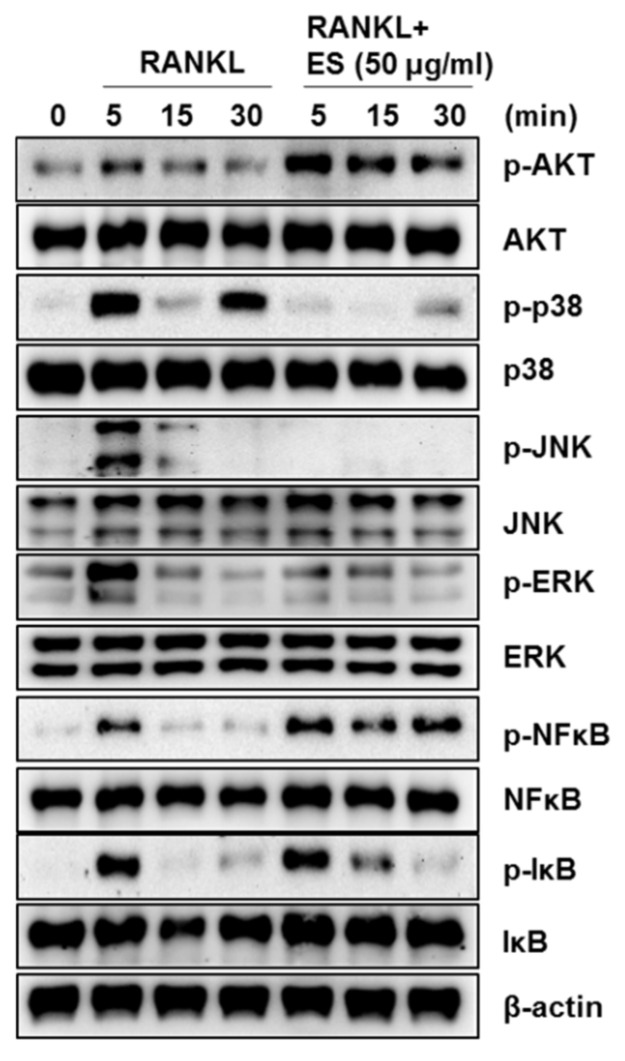
ES suppressed RANKL-stimulated mitogen-activated protein kinases (MAPKs) and nuclear factor kappa-B (NF-κB) signaling. BMMs were pretreated with or without ES (50 μg/mL) for 1 h in α-MEM containing M-CSF (30 ng/mL) and then treated RANKL (100 ng/mL) for the indicated times. The cell lysates were subjected to western blot analysis with the indicated antibodies. β-Actin was used as the internal control.

Based on the findings that ES inhibited osteoclast formation and bone resorption, we investigated the effect of ES on RANKL-mediated early signaling, focusing on Akt, MAPKs (p38, JNK, and ERK), and NF-κB. In response to RANKL stimulation, the levels of phosphorylated p38, JNK, ERK, and Akt peaked at 5 min. ES inhibited RANKL-activated phosphorylation of p38, JNK, and ERK; however, it did not exert any effect on RANKL-induced Akt phosphorylation (Figure 6). NF-κB is an important transcription factor and is stimulated by RANKL during osteoclast differentiation. Therefore, using Western blotting, we investigated whether ES inhibited osteoclast differentiation via the suppression of the NF-κB signaling pathway. As shown in Figure 6, RANKL-induced phosphorylation of NF-κB and IκB was not affected by the treatment with ES. These results demonstrated that ES suppressed RANKL-activated osteoclast differentiation by attenuating p38, JNK, and ERK phosphorylation.

### 2.6. ES Attenuates OVX-Mediated Bone Loss

**Figure 7:**
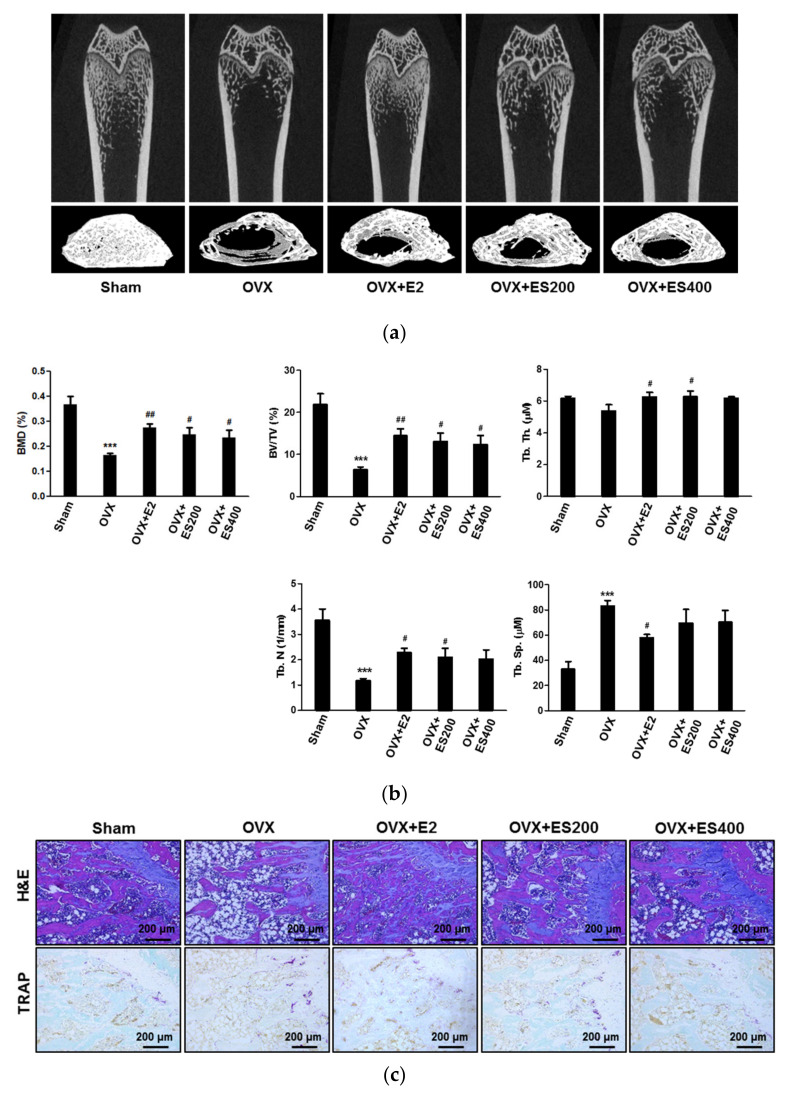
ES prevented ovariectomy (OVX)-mediated bone loss. SD rats (8-week-old) were ovariectomized at 7 weeks old. ES (200 and 400 mg/kg) or PBS was administered orally once a day for 12 weeks. After 12 weeks, all rats were euthanized. (**a**) Representative three-dimensional (3D) reconstruction images of femurs from micro-CT. (**b**) Bone mineral density (BMD), bone volume/tissue volume (BV/TV), trabecular thickness (Tb.Th.), trabecular number (Tb.N.), and trabecular separation (Tb.Sp.) of the femurs were determined and analyzed by Skyscan 1076 software. (**c**) Decalcified femurs were embedded in paraffin and sectioned. Sections were stained with hematoxylin and eosin (H&E) (top) and with TRAP (bottom). *n* = 6 rats/group *** *p* < 0.001 vs. the Sham group; ^#^ *p* < 0.05 and ^##^ *p* < 0.01 vs. the OVX group.

To investigate whether ES-mediated osteoclast inhibition in vitro could also block bone loss in vivo, ovariectomized 8-week-old SD rats were treated with ES for 12 weeks. After 12 weeks, the femurs of the sacrificed rats were used for micro-CT and histological analysis. Micro-CT images showed that the OVX group, as expected, had significant trabecular bone loss; however, the treatment with ES partially recovered the OVX-induced bone loss (Figure 7a). Furthermore, BMD was markedly lower in the OVX group than in the sham group (Figure 7b). BMD in the groups treated with ES (200 and 400 mg/kg) for 12 weeks was markedly higher than that in the OVX group. Moreover, the microstructural parameter analysis of femurs showed that BV/TV, Tb.N., and Tb.Th. decreased, while Tb.Sp. was increased in the OVX group. These changes were significantly reversed in ES (200 and 400 mg/kg)-treated groups (Figure 7b). This effect was similar to the results observed in the E_2_ group. Consistent with the micro-CT results, histological analysis revealed that ES treatment partially reversed OVX-mediated trabecular bone loss and attenuated the formation of TRAP-positive cells (Figure 7c).

## 3. Discussion

Bone homeostasis is an active process involving osteoclastic bone resorption and osteoblastic bone formation. An imbalance in bone homeostasis leads to excessive bone resorption by osteoclasts, resulting in various bone-related diseases, such as osteoporosis and rheumatoid arthritis (RA) [25]. Osteoclasts, which are the only bone-resorbing cells, are multinucleated cells derived from hematopoietic stem cells of the monocyte or macrophage lineage [26]. Osteoclast differentiation is regulated by the sequential expression of various genes and is controlled by two cytokines, M-CSF and RANKL. M-CSF is a hematopoietic growth factor involved in the survival, growth, differentiation, and function of osteoclast precursors. The key function of M-CSF in osteoclast differentiation was revealed by studies showing that macrophage-deficient osteopetrotic (*op/op*) mice have a severe osteoclast deficiency [27].

RANKL is expressed by osteoblasts, osteocytes, and immune cells, and is another important factor for osteoclast differentiation [28]. RANKL binds to its receptor RANK on the surface of osteoclast precursors and then activates tumor necrosis factor receptor-associated factor 6 (TRAF6) and, subsequently, MAPKs and the NF-κB signaling pathway [29,30]. MAPKs, including p38, ERK, and JNK, are involved in the regulation of osteoclast differentiation and play critical roles in osteoclast fusion, migration, adhesion, and osteoclastic bone resorption [31]. NF-κB is one of the key transcription factors, and NF-κB p50^−/−^p52^−/−^ mice are osteopetrotic due to blocked osteoclast differentiation [32]. RANKL-induced MAPKs and NF-κB activate c-Fos and NFATc1. c-FOS, a member of the activator protein-1 (AP-1) family, is induced at an early stage during osteoclast differentiation [33]. Mice lacking c-Fos develop osteopetrosis due to defects in osteoclast differentiation [34]. NAFTc1, another transcription factor activated by RANKL, plays an essential role in osteoclast formation and function. For example, NFATc1-deficient embryonic stem cells cannot differentiate into osteoclasts [35] while NFATc1-deficient mice exhibit a severe osteopetrotic phenotype [36]. Moreover, NFATc1 overexpression has been shown to restore osteoclastogenesis in precursors lacking c-Fos [37]. c-Fos is fundamental for the RANKL-induced activation of NFATc1, and these two transcription factors are functionally linked. At the late stage of RANKL-RANK signaling, NFATc1 induces various osteoclast marker genes, such as TRAP, OSCAR, MMP-9, calcitonin receptor, cathepsin K, Atp6v0d2, DC-STAMP, and OC-STAMP, which are indispensable for osteoclast differentiation, fusion, and function [38,39,40,41,42].

ES has been used in clinical applications to treat bone-related disease for a long time [43]. Based on these effects, many studies have consistently demonstrated scientific evidence to support the use of ES. According to Xu et al. [44], *Acanthopanax senticosus*, one of the *Eleutherococcus* species, has been shown to suppress RANKL-stimulated osteoclastogenesis by reducing NFATc1, c-Fos, and the expression of osteoclastogenesis-related genes, including c-Src, β3-integrin, cathepsin K, TRAP, and MMP-9, in RAW 264.7 cells. These authors showed the protective effect of *Acanthopanax senticosus* on ovariectomy-induced bone loss in middle-aged mice. Moreover, Zhang et al. [21] reported that ES prevented OVX-mediated bone loss in Wistar rats, by attenuating bone resorption via the downregulation of RANKL expression in the tibia. ES administration significantly reduced the level of IL-1β and increased the level of CT in serum. However, the inhibitory effects of ES on RANKL-activated osteoclast differentiation in primary mouse BMMs and the molecular mechanism underlying its activity remain unclear.

In the present study, the anti-osteoclastogenesis effect of ES was demonstrated in primary mouse BMMs. First, we found that ES substantially abrogated RANKL-activated osteoclast differentiation, bone resorption, and F-actin belt formation. ES suppressed the MAPK signaling pathway by decreasing the phosphorylation of p-38, JNK, and ERK but did not affect the phosphorylation of Akt, NF-κB, and I-κB. In addition, ES effectively abrogated osteoclast differentiation via the inhibition of c-Fos and NFATc1, which subsequently attenuated the expression of downstream osteoclast-specific genes.

OVX is a well-established animal model to study osteoporosis, as it leads to severe bone loss as the result of estrogen deficiency [45]. Therefore, to investigate the effects of ES on osteoclastogenesis in vivo, we decided to use this model of bone loss. Our results for BMD, BV/TV, Tb.Th, Tb.N, and Tb.Sp showed that ES prevented OVX-induced bone loss. Histological analysis further confirmed that ES reduced OVX-mediated loss of trabecular bone, as well as the number of TRAP-positive osteoclasts.

Taken together, the results of this study demonstrated the anti-osteoporotic effects of ES on RANKL-activated osteoclast differentiation and OVX-mediated bone loss. These findings will help deepen our understanding of the pharmacological effects of ES and facilitate its utilization in the treatment of osteoporosis.

## 4. Materials and Methods

### 4.1. Reagents

Human RANKL and M-CSF were obtained from Peprotech EC Ltd. (London, UK). Akt, phospho-Akt, p38, phospho-p38, JNK, phospho-JNK, ERK, phospho-ERK, IκB, and phospho-IκB were purchased from Cell Signaling Technology Inc. (Beverly, MA, USA). GSK3β, phospho-GSK3β, and Runx2 antibodies were purchased from Bioworld Technology Inc. (St. Louis. Park, MN, USA). NFATc1, c-Fos, phospho-Smad, Smad, and β-actin antibodies were obtained from Santa Cruz Biotechnology (Santa Cruz, CA, USA).

### 4.2. Preparation of ES

The root barks of *Eleutherococcus sessiliflorus* were purchased from Humanherb (Daegu, Korea) in accord with the good manufacturing practices (GMP) procedures certified by the Korea Food and Drug Administration (KFDA) and authenticated by Professor Guem-San Lee (Department of Herbology, Wonkwang University School of Korean Medicine). Voucher specimen (No. WP-2018-04) was deposited at the College of Pharmacy, Wonkwang University. Coarse-cut ES root barks (100 g) were placed in distilled water for 30 min and then extracted by heating at 100 °C for 2 h. The extract was filtered using filter paper (110 mm, Advantec No.2), and the filtrate was concentrated at 60 °C using a rotary evaporator (Buchi Labortechnik AG, Flawil, Switzerland). The filtrate was further lyophilized using a freeze dryer into a dry powder. The ES extract powder (yield: 8.4%) were deposited in the College of Pharmacy, Wonkwang University for future.

### 4.3. HPLC Analysis

Agilent 1200 series HPLC system (Agilent Technologies, Wilmington, DE, USA) consisted of a quaternary pump VL (G7111A), autosampler (G7129A), ICC column oven heater (G7129A), and variable wavelength detector (G7114A). For HPLC detection of the eleutheroside B and protocatechuic acid, an Agilent SB-C18 HPLC column (4.6 × 250 mm, 5 μm, Zorbax, Agilent, Santa Clara, CA, USA) was used. The separating conditions of eleutheroside B were as follows: methanol: water: phosphoric acid (20:80:0.1, *v/v/v*), and flow rate, 1.0 mL min^−1^. The chromatograms were recorded at 265 nm by monitoring spectra within a wavelength range of 190 to 600 nm. The separating conditions of protocatechuic acid were as follows: methanol: water: phosphoric acid (13:87:0.1, *v/v/v*), and flow rate, 1.0 mL min^−1^. The chromatograms were recorded at 260 nm.

### 4.4. Isolation of BMMs

The protocol for animal use in this experiment was approved by the guidelines of the Institute Committee of Wonkwang University (Approval number: WKU20-20). The animals were housed under conditions of controlled temperature (22 ± 1 °C) with 12 h light and 12 h dark cycles. Male 6-week-old ICR mice were obtained from Samtako Bio Korea (Osan, Korea). Bone marrow cells (BMCs) were isolated from tibias and femurs of ICR mice by flushing with α-MEM (Gibco BRL, Grand Island, NY, USA) containing 1% antibiotics; red blood cells were lysed using the RBC lysis buffer. After culturing for 3 days, the floating cells were removed by aspiration and adherent cells were used as BMMs.

### 4.5. Osteoclast Differentiation and TRAP Assay

BMMs (1 × 10^4^ cells/well) were differentiated for 4 days with M-CSF (30 ng/mL) and RANKL (100 ng/mL) in the presence or absence of ES. Cells were washed with PBS, fixed with 3.7% formalin, permeabilized with 0.1% Triton X-100, and then stained with the TRAP solution (1 mg/mL fast red violet LB, 100 µg/mL naphthol AS-MX phosphate, 0.1 M sodium acetate [pH 5.0], 50 mM sodium tartrate). A number of TRAP-positive multinucleated osteoclasts (≥3 nuclei) per cell were counted as osteoclasts. Images were acquired using a microscope (Leica, Wetzlar, Germany). To evaluate total TRAP activity, cells were incubated with the lysis buffer (1% Triton X-100 in TRAP buffer [50 mM sodium tartrate and 0.1 M sodium acetate, pH 5.2]) for 10 min and incubated with substrate solution (1 mg/mL p-nitrophenyl phosphate [pNPP] in TRAP buffer) for 30 min at 37 °C. The absorbance was measured using a microplate reader at 405 nm (Molecular Devices, Sunnyvale, CA, USA).

### 4.6. F-actin Belts Staining

BMMs were cultured for 4 days as described in the previous section. After 4 days, the cells were fixed with PBS containing 4% paraformaldehyde for 15 min and permeabilized using 0.1% Triton X-100 for 20 min. Cells were blocked with 0.25% BSA/0.1% azide in PBS for 1 h. Cells were stained with Texas Red™-X phalloidin (Molecular Probes, Eugene, OR, USA) and DAPI (0.1 µg/mL). Images were acquired using a fluorescent microscope (EVOS, Thermo Fisher Scientific, New York, NY, USA).

### 4.7. Cell Viability

BMMs (1 × 10^4^ cells/well) were seeded in a 96-well microplate with α-MEM supplemented with M-CSF (30 ng/mL). After 24 h of incubation, cells were treated with various concentrations of ES and then incubated for 4 days. Cell viability was performed using the XTT kit according to the manufacturer’s protocol (Roche, Mannheim, Germany).

### 4.8. Bone Resorption Assay

Primary osteoblastic cells were isolated from calvaria of neonatal ICR mice (1-day-old). BMCs and primary osteoblasts were co-cultured on collagen gel-coated culture dishes with α-MEM supplemented 10^−8^ M 1,25-dihydroxyvitamin D_3_ (VitD_3_) and 10^−6^ M prostaglandin E2 (PGE2) for 7 days. Mature osteoclasts were detached using 0.1% collagenase and then seeded into Osteo assay surface 96-well plates (Corning, New York, NY, USA) with or without ES in the presence of M-CSF (30 ng/mL) and RANKL (100 ng/mL). After 24 h of incubation, cells were removed using 10% NaClO. Images were determined using a light microscope and the total area of resorption pits was quantified using ImageJ 1.50i software.

### 4.9. Western Blot Analysis

After treatments, cells were harvested and lysed with the ice-cold lysis buffer (Biosesang, Seongnam, Korea) containing Na_3_VO_4_ and a protease inhibitor cocktail (Sigma-Aldrich, St.Louis, MO, USA). The concentration of total protein was evaluated using a BCA Protein Assay Kit (Thermo Scientific). Proteins (20–30 µg) were separated by 10% sodium dodecyl sulfate-polyacrylamide gel electrophoresis and transferred onto polyvinylidene difluoride membranes (Bio-Rad, Hercules, CA, USA). The membranes were blocked with 5% BSA and then probed with primary antibodies (1:1000). These antibodies were detected using HRP-conjugated secondary antibodies (1:5000). Chemiluminescent signals were visualized using the Western ECL solution (Bio-Rad, Hercules, CA, USA) and FlourChem E system (ProteinSimple, San Jose, CA, USA).

### 4.10. Real-Time RT-PCR Analysis

Total RNA was isolated using the Isol-RNA lysis reagent (PRIME, Gaithersburg, MD, USA). cDNA was synthesized from 1 pg of total RNA using the ReverTra Ace qPCR RT Kit (Toyobo, Osaka, Japan) according to the manufacturer’s instructions. Real-time RT-PCR was performed using a SYBR^®^ Green Realtime PCR Master Mix (Toyobo, Osaka, Japan) and Step-One Plus™ RT-PCR system (Applied Biosystems, Foster City, CA, USA). Target gene expression levels were normalized to the expression of endogenous GAPDH gene and calculated from the cycle threshold (Ct) value using the 2^-ΔΔCt^ method. Primers (Bioneer, Daejeon, Korea) used for real-time RT-PCR are listed in Table 1.

### 4.11. Animal Bone Loss Model

Female Sprague–Dawley (SD rats, 7 weeks old, 130–170 g) rats were used in these experiments. After 1 week of acclimatization, the rats were anesthetized with a ketamine-xylazine mixture (5:1, I.P.), and the ovaries were removed bilaterally. One week after surgery, rats were randomly divided into five groups (*n* = 6 rats/group) as follows: (1) sham-operated rats (Sham); (2) ovariectomized rats (OVX); (3) OVX rats treated with 17β-estradiol (E_2_, 100 µg/kg once a day, S.C.); (4) OVX rats treated with low doses of ES (200 mg/kg, P.O.); and (5) OVX rats treated with high doses of ES (400 mg/kg, P.O.). As osteoporosis induced by ovariectomy is associated with estrogen deficiency, E2 treatment was used as a positive control. ES or PBS was administered orally once a day for 12 weeks. The rats were euthanized at 12 weeks by diethyl ether inhalation, and femurs were dissected out for micro-CT (Skyscan 1076, Bruker, Kontich, Belgium) and histological analysis.

### 4.12. Micro-CT and Histological Analysis

The femurs were examined using a Skyscan 1076 scanner at a resolution of 35 μm. The X-ray source was set at an accelerating voltage of 50 kV and a beam current of 100 μA with a 0.5 mm aluminum (Al) filter. Raw scan images were reconstructed using Skyscan NRecon software (ver. 1.6.10.1, Bruker, Kontich, Belgium). Reconstructed images were segmented to allow trabecular bone structure quantification using CTAn software (ver. 1.18.4.0, Bruker, Kontich, Belgium). 3D image visualization was performed using Ant software (ver. 2.4; Bruker, Kontich, Belgium). The femurs were fixed in 4% paraformaldehyde for 1 day and decalcified in 12% EDTA (pH 7.4) for 3 weeks. Femurs were then embedded in paraffin. The blocks of embedded femurs were cut into 5 μm serial sections using a microtome (RM2125, Leica Microsystems, Bannockburn, IL, USA) and then subjected to H&E and TRAP staining.

### 4.13. Statistical Analysis

All data are expressed as the mean ± standard deviation (S.D.), representing the results of more than three independent experiments. Statistical analyses were performed using the SPSS software, version 12.0 (SPSS Inc., Chicago, IL, USA). Significant differences were analyzed using the one-way ANOVA followed by LSD post hoc analysis. *p*-Values < 0.05 were considered statistically significant.

## Figures and Tables

**Table 1:** Primer sequences used for real-time RT-PCR analysis.

Target Gene		Primer Sequence (5′-3′)
c-Fos	Forward	CTGGTGCAGCCCACTCTGGTC
	Reverse	CTTTCAGCAGATTGGCAATCTC
NFATc1	Forward	CAACGCCCTGACCACCGATAG
	Reverse	GGCTGCCTTCCGTCTCATAGT
TRAP	Forward	ACTTCCCCAGCCCTTACTAC
	Reverse	TCAGCACATAGCCCACACCG
OSCAR	Forward	CTGCTGGTAACGGATCAGCTCCCCAGA
	Reverse	CCAAGGAGCCAGAACCTTCGAAACT
Atp6v0d2	Forward	TCAGATCTCTTCAAGGCTGTGCTG
	Reverse	GTGCCAAATGAGTTCAGAGTGATG
Cathepsin K	Forward	ACGGAGGCATTGACTCTGAAGATG
	Reverse	GTTGTTCTTATTCCGAGCCAAGAG
MMP-9	Forward	TCCAACCTCACGGACACCC
	Reverse	AGCAAAGCCGGCCGTAGA
Calcitonin receptor	Forward	TCCAACAAGGTGCTTGGGAA
	Reverse	CTTGAACTGCGTCCACTGGG
DC-STAMP	Forward	TCCTCCATGAACAAACAGTTCCA
	Reverse	AGACGTGGTTTAGGAATGCAGCTC
OC-STAMP	Forward	ATGAGGACCATCAGGGCAGCCACG
	Reverse	GGAGAAGCTGGGTCAGTAGTTCGT
GAPDH	Forward	ACCACAGTCCATGCCATCAC
	Reverse	TCCACCACCCTGTTGCTGTA

## Data Availability

The data presented in this study are available within this article.

## Sample Availability

Samples of the ES extracts are available from the authors.
